# Striatal Tyrosine Hydroxylase Is Stimulated via TAAR1 by 3-Iodothyronamine, But Not by Tyramine or β-Phenylethylamine

**DOI:** 10.3389/fphar.2018.00166

**Published:** 2018-03-01

**Authors:** Xiaoqun Zhang, Ioannis Mantas, Alexandra Alvarsson, Takashi Yoshitake, Mohammadreza Shariatgorji, Marcela Pereira, Anna Nilsson, Jan Kehr, Per E. Andrén, Mark J. Millan, Karima Chergui, Per Svenningsson

**Affiliations:** ^1^Section of Translational Neuropharmacology, Department of Clinical Neuroscience, Center for Molecular Medicine L8:01, Karolinska Institute, Karolinska University Hospital, Stockholm, Sweden; ^2^Section of Pharmacological Neurochemistry, Department of Physiology and Pharmacology, Karolinska Institute, Solna, Sweden; ^3^Biomolecular Mass Spectrometry Imaging, National Resource for Mass Spectrometry Imaging, Science for Life Laboratory, Department of Pharmaceutical Biosciences, Uppsala University, Uppsala, Sweden; ^4^Centre for Therapeutic Innovation-CNS, Institut de Recherches Servier, Centre de Recherches de Croissy, Paris, France; ^5^Section of Molecular Neurophysiology, Department of Physiology and Pharmacology, Karolinska Institute, Solna, Sweden

**Keywords:** trace amine-associated receptor 1, tyrosine hydroxylase, evoked dopamine release, tyramine, T_1_AM

## Abstract

The trace amine-associated receptor 1 (TAAR1) is expressed by dopaminergic neurons, but the precise influence of trace amines upon their functional activity remains to be fully characterized. Here, we examined the regulation of tyrosine hydroxylase (TH) by tyramine and beta-phenylethylamine (β-PEA) compared to 3-iodothyronamine (T_1_AM). Immunoblotting and amperometry were performed in dorsal striatal slices from wild-type (WT) and TAAR1 knockout (KO) mice. T_1_AM increased TH phosphorylation at both Ser^19^ and Ser^40^, actions that should promote functional activity of TH. Indeed, HPLC data revealed higher rates of L-dihydroxyphenylalanine (DOPA) accumulation in WT animals treated with T_1_AM after the administration of a DOPA decarboxylase inhibitor. These effects were abolished both in TAAR1 KO mice and by the TAAR1 antagonist, EPPTB. Further, they were specific inasmuch as Ser^845^ phosphorylation of the post-synaptic GluA1 AMPAR subunit was unaffected. The effects of T_1_AM on TH phosphorylation at both Ser^19^ (CamKII-targeted), and Ser^40^ (PKA-phosphorylated) were inhibited by KN-92 and H-89, inhibitors of CamKII and PKA respectively. Conversely, there was no effect of an EPAC analog, 8-CPT-2Me-cAMP, on TH phosphorylation. In line with these data, T_1_AM increased evoked striatal dopamine release in TAAR1 WT mice, an action blunted in TAAR1 KO mice and by EPPTB. Mass spectrometry imaging revealed no endogenous T_1_AM in the brain, but detected T_1_AM in several brain areas upon systemic administration in both WT and TAAR1 KO mice. In contrast to T_1_AM, tyramine decreased the phosphorylation of Ser^40^-TH, while increasing Ser^845^-GluA1 phosphorylation, actions that were not blocked in TAAR1 KO mice. Likewise, β-PEA reduced Ser^40^-TH and tended to promote Ser^845^-GluA1 phosphorylation. The D_1_ receptor antagonist SCH23390 blocked tyramine-induced Ser^845^-GluA1 phosphorylation, but had no effect on tyramine- or β-PEA-induced Ser^40^-TH phosphorylation. In conclusion, by intracellular cascades involving CaMKII and PKA, T_1_AM, but not tyramine and β-PEA, acts *via* TAAR1 to promote the phosphorylation and functional activity of TH in the dorsal striatum, supporting a modulatory influence on dopamine transmission.

## Introduction

Classical trace amines (TAs), including tyramine, beta-phenylethylamine (β-PEA), tryptamine and octopamine, have been implicated in a number of neuropsychiatric disorders associated with monoaminergic dysfunction, including schizophrenia, major depression, and Parkinson’s disease (Boulton, 1980; Premont et al., 2001; Branchek and Blackburn, 2003; Burchett and Hicks, 2006; Berry, 2007; Sotnikova et al., 2009; Millan, 2014; Khan and Nawaz, 2016; Berry et al., 2017). TAs are structurally, metabolically and functionally related to monoamines, and are synthesized in nerve terminals by decarboxylation of the amino acids that serve as precursors for dopamine (DA), noradrenaline, and serotonin (Berry, 2007). TAs are present in mammalian tissues at very low (nanomolar) concentrations (Grandy, 2007), and are stored in monoaminergic nerve terminals where they are released together with monoamines (Branchek and Blackburn, 2003). TAs are recognized as substrates for monoamine transporters, suggesting similarities between the regulation of extracellular levels of TAs and monoamines (Meiergerd and Schenk, 1994; Burnette et al., 1996; Sitte et al., 1998; Li et al., 2002; Miller et al., 2005). Neuroanatomical observations and cellular studies indicate that TAs have a modulatory influence on monoaminergic neurotransmission, in particular on dopaminergic transmission, which is expressed across multiple cerebral structures (Federici et al., 2005; Xie and Miller, 2007, 2008; Sotnikova et al., 2008; Khan and Nawaz, 2016; Millan et al., 2016). A reduction in TA levels has been proposed to be associated with depressed states (Sabelli and Mosnaim, 1974; Sandler et al., 1979; Davis and Boulton, 1994; Sabelli et al., 1996; Szabo et al., 2001; Branchek and Blackburn, 2003). TA levels are enhanced by inhibition of monoamine oxidase A and B in animals where the corresponding genes have been deleted (Holschneider et al., 2001).

For a long time the pharmacological effects of TAs were attributed to a direct interference with aminergic pathways, up until the cloning and characterization of a large family of G protein-coupled receptors, named trace amine-associated receptors (TAARs) which were found to be activated by TAs (Borowsky et al., 2001; Bunzow et al., 2001). These receptors responded to the endogenous TAs along with several amphetamines. Outside the central nervous system (CNS), TAAR1 is expressed in pancreatic β-cells, stomach, intestines, thyroid gland, and leukocytes (Szumska et al., 2015; Khan and Nawaz, 2016; Berry et al., 2017). It is therefore interesting that endogenous 3-iodothyronamine (T_1_AM), which is a derivative of thyroid hormone (thyroxine, or T_4_), has been found to be an endogenous agonist at TAAR1 (Scanlan et al., 2004; Hart et al., 2006; Doyle et al., 2007). The reduction of core temperature and cardiac output induced by T_1_AM, which contrast to the effects induced by thyroxine itself, have been suggested to be mediated by TAAR1 activation (Scanlan et al., 2004; Zucchi et al., 2006; Chiellini et al., 2007; Doyle et al., 2007; Ghelardoni et al., 2009; Di Cara et al., 2011). In the brain, TAAR1 is enriched in the major nuclei of the monoaminergic system such as the ventral tegmental area (VTA), substantia nigra pars compacta (SNc), locus coeruleus and raphe nuclei as well as their projection targets, the hypothalamus, layer V pyramidal neurons of prefrontal cortex (PFC), caudate nucleus, putamen, nucleus accumbens (NAc), hippocampus, and amygdala (Borowsky et al., 2001; Bunzow et al., 2001; Wolinsky et al., 2007; Lindemann et al., 2008; Espinoza et al., 2015b; Khan and Nawaz, 2016; Pei et al., 2016; Berry et al., 2017). However, the highest TAAR1 mRNA levels are clearly found in the dopaminergic cell groups (VTA and SNc) as compared to other brain regions (Liu et al., 2017). The detailed expression pattern of TAAR1 among the different neuronal populations has not been yet fully defined. Nonetheless, since its discovery, TAAR1 has emerged as a modulator of monoaminergic functions and a mediator of psychostimulant effects (Miller et al., 2005; Xie and Miller, 2009; Di Cara et al., 2011).

Trace amine-associated receptor 1 is coupled with stimulatory G_s_ proteins, but its signaling also involves the G protein independent β-arrestin2/Akt/Glucogen Synthase Kinase-3β (GSK-3β) pathway (Harmeier et al., 2015). The latter pathway is known to be downstream of D_2_ receptors (Espinoza et al., 2011; Pei et al., 2016). There is evidence that TAAR1 interacts directly with D_2_ receptor by forming heterodimers, however, a peculiar aspect of the receptor is its intracellular residence (Pei et al., 2016). This intracellular localisation of the receptor has been indicated by experiments with tagged TAAR1 proteins where it was observed that the chimeric molecules showed robust intracellular distribution (Bunzow et al., 2001; Xie et al., 2007; Harmeier et al., 2015).

The elucidation of TAAR1 function has been greatly facilitated by the development of selective pharmacological tools and the generation of mutant TAAR1 animal models. *N*-(3-ethoxy-phenyl)-4-pyrrolidin-1-yl-3-trifluoromethyl-benzamide (EPPTB) is a selective TAAR1 antagonist (Bradaia et al., 2009; Stalder et al., 2011), whereas several agonists, including RO5166017, binds to TAAR1 with high affinity (Revel et al., 2011). TAAR1 knockout (KO) mouse lines have been generated to further delineate the role of TAAR1 (Wolinsky et al., 2007; Lindemann et al., 2008; Di Cara et al., 2011). There are no gross behavioral abnormalities in TAAR1 KO mice, but upon closer examination they show an impulsive and perseverative phenotype (Wolinsky et al., 2007; Espinoza et al., 2015a). Baseline extracellular DA levels in striatum are similar between wild-type (WT) and TAAR1 KO mice (Lindemann et al., 2008; Di Cara et al., 2011; Lam et al., 2015; Pei et al., 2016). However, electrophysiological experiments have shown that dopamine neurons in VTA and serotonin neurons in dorsal raphe nucleus (DRN) from TAAR1 KO mice display increased firing rates compared with WT mice (Lindemann et al., 2008; Bradaia et al., 2009; Miller, 2011). The endogenous TA, tyramine, specifically decreased the spike frequency of VTA neurons in WT but not in TAAR1 KO mice (Lindemann et al., 2008).

Trace amine-associated receptor 1 KO mice have repeatedly been shown to display increased sensitivity to amphetamines, measured as an enhanced increase in locomotor activity and enhanced striatal release of DA compared with WT animals (Wolinsky et al., 2007; Lindemann et al., 2008; Miller, 2011; Achat-Mendes et al., 2012). Accordingly, TAAR1 is thought to act in the mesocorticolimbic system to regulate cocaine-seeking behavior (Liu et al., 2017). We have also found an increased behavioral responsivity toward L-dihydroxyphenylalanine (L-DOPA) in TAAR1 KO mice rendered unilaterally dopamine denervated by 6-hydroxydopamine injections in the median forebrain bundle (Alvarsson et al., 2015). Taken together, studies in TAAR1 KO animals support the role of TAAR1 as a regulator of dopaminergic neurotransmission, underlining the role of TAAR1 as a potential novel target for the treatment of neuropsychiatric disorders.

Here we extended the studies of TAs, thyronamines and TAAR1 on dopamine neurotransmission in the dorsal striatum. Special emphasis was put on T_1_AM, which contains the aryethylamine backbone of monoamine neurotransmitters (Chiellini et al., 2017). Remarkably, T_1_AM is a product of the enzymatic deiodination and decarboxylation of T_4_ (Hoefig et al., 2016). We used slices from WT and TAAR1 KO mice and examined the effects of tyramine, β-PEA, and T_1_AM on the phosphorylation state of tyrosine hydroxylase (TH), which regulates DA synthesis (Daubner et al., 2011), along with TH activity. For further evaluation of TH activity, we measured with high pressure liquid chromatography (HPLC) the levels of L-DOPA after the administration of a DOPA decarboxylase inhibitor. Using the same slices, we also studied effects of T_1_AM on evoked DA release. We also studied effects on the phosphorylation state of the post-synaptic alpha-amino-3-hydroxy-5-methyl-4-isoxazolepropionic acid (AMPA) receptor subunit GluA1, which plays a crucial role in regulating transmission and plasticity at excitatory synapses in striatum. Finally, mass spectrometry imaging was used to detect T_1_AM at baseline and upon systemic administration.

## Materials and Methods

### Animals

The experiments were approved by the local ethical committee at Karolinska Institute (N351/08) and conducted in accordance with the European Communities Council Directive of 24 November 1986 (86/609/EEC). Adult male WT and TAAR1 KO mice on a C57Bl6 background were used (Di Cara et al., 2011). They were housed in temperature- and humidity-controlled rooms (20°C, 53% humidity) with a 12 h dark/light cycle. They had access to standard lab pellets and water *ad libitum*.

### Preparation and Incubation of Dorsal Striatal Slices for Phosphorylation Experiments

Mouse brains were rapidly removed and placed in ice-cold, oxygenated (95% O_2_/5% CO_2_) artificial cerebrospinal fluid (aCSF) containing (in mM): 126 NaCl, 2.5 KCl, 1.2 NaH_2_PO_4_, 1.3 MgCl_2_, 2.4 CaCl_2_, 10 glucose and 26 NaHCO_3_, pH 7.4. Coronal slices (300 μm thick) were prepared using a Leica vibratome (Leica, Wetzlar, Germany). Dorsal striata were dissected from the slices in ice-cold aCSF buffer. Each slice was placed in a polypropylene incubation tube with 2 ml fresh aCSF buffer. The slices were preincubated at 30°C under constant oxygenation (95% O_2_/5% CO_2_) for 60 min with a change of buffer after 30 min. The buffer was then replaced with fresh aCSF and slices were treated with tyramine (1, 10, 100 μM; Sigma-Aldrich, St. Louis, MO, United States), T_1_AM (1, 10, 100 μM; synthesized by Servier, kind gift from Mark J. Millan), β-PEA (100 μM; Sigma-Aldrich), SCH23390 (5 μM; Sigma-Aldrich), EPPTB (10 nM, synthesized by Servier, kind gift from Mark J. Millan), KN-92 (10 μM; Sigma-RBI), H-89 (10 μM; Calbiochem, Gibbstown, NJ, United States) and 8-CPT-2Me-cAMP (10 μM; Tocris Bioscience, Bristol, United Kingdom), alone or in combination. The higher doses of all compounds exceeds by far the IC_50_ or Kd values for their respective target, but it is known that much higher concentrations are needed to exert actions in brain slices when compared to cell culture systems (Nishi et al., 1997). After the drug treatment, the buffer was removed, the slices rapidly frozen on dry ice and stored at -80°C until assayed.

### Immunoblotting

Immunoblotting was performed as described earlier (Qi et al., 2009). Frozen tissue samples were sonicated in 1% SDS, transferred to Eppendorf tubes and boiled for additional 10 min. Small aliquots of the homogenate were retained for protein determination using the bicinchoninic acid protein assay method (Pierce, Rockford, IL, United States). Equal amounts of protein (20 μg) were loaded onto 12% acrylamide gels, and the proteins were separated by SDS-PAGE and transferred to Immobilon^®^-P Polyvinylidene Difluoride membranes (Sigma). Immunoblotting was performed on the membranes using P-Ser^19^-TH (Merck Millipore, Billerica, MA, United States), P-Ser^31^-TH (Millipore), P-Ser^40^-TH (Millipore), P-Ser^845^-GluA1 (UBI), and antibodies, which are not phosphorylation state-specific to estimate total levels of TH (Millipore) and GluA1 (UBI). The antibody binding was detected by incubation with goat anti-mouse or anti-rabbit horseradish peroxidase-linked IgG (1:6000–8000 dilution) and detected using ECL immunoblotting detection reagents (GE Healthcare, Little Chalfont, United Kingdom).

### Determination of L-DOPA in Dorsal Striatal Slices

Dorsal striatal slices were incubated for 5 min with T_1_AM (10 μM) or tyramine (100 μM), and then for 15 min with T_1_AM or tyramine along with the L-amino acid decarboxylase inhibitor NSD-1015 (100 μM, Sigma-Aldrich). After the removal of the solutions, tissue slices were frozen and sonicated (10,000 *g* for 10 min) in 100 μL perchloric acid (0.1 mM). The pellets were resuspended in 100 μl 1% sodium dodecyl sulfate and the protein content was determined. The level of L-DOPA in the supernatant was determined using HPLC coupled to an electrochemical detection system with a refrigerated microsampling unit (model CMA/200; CMA Microdialysis, Kista, Sweden). The HPLC apparatus comprised an HPLC pump (model 2150; Pharmacia LKB Biotechnology AB, Uppsala, Sweden) that kept a constant flow of 0.2 mL/min of the mobile phase (0.12 m NaH_2_PO_4_H_2_O; 0.09 m EDTA, 0.05 mm 1-octanesulfonic acid, and 15% methanol, pH 4.2) and a pressure of ∼0.5 bar on a reverse-phase ion pair C-18 column prepacked with Biophase ODS 5 μm particles (BAS, West Lafayette, IN, United States). L-DOPA was detected with an amperometric detector (model LC-4C; BAS) and a glassy carbon electrode set at 0.75 V. The limit of detection was ∼10 nM.

### Amperometry in Dorsal Striatal Slices

Sagittal striatal brain slices were prepared and maintained as above. Amperometric detection of DA release was performed as described earlier (Zhang et al., 2014a). Carbon fiber electrodes (10 μm in diameter, World Precision Instruments, Hertfordshire, England) had an active part (100 μm) that was positioned within the dorsal striatum in the brain slice. A constant voltage of +500 mV was applied to the carbon fiber via an Axopatch 200B amplifier (Axon Instruments) and currents were recorded with the same amplifier. A stimulating electrode (patch electrode filled with aCSF) was placed on the slice surface, in the vicinity of the carbon fiber electrode. Stimulation consisted of a single pulse (0.1 ms, 8–14 μA) applied every minute, which evoked a response corresponding to oxidation of DA at the surface of the electrode. When the carbon fiber electrode was held at 0 mV, stimulation of the slice did not produce any current.

### Matrix-Assisted Laser Desorption Ionization (MALDI) – Mass Spectrometry (MS) Imaging

Adult male WT and TAAR1 KO mice were injected with saline or T_1_AM (20 mg/kg, i.p.) and killed by decapitation 30 or 60 min post-dose. All brains were immediately removed, snap frozen, and stored at -80°C until further analysis. The frozen brain tissues were cut using a cryostat-microtome (Leica CM3050S; Leica Microsystems, Welzlar, Germany) at a thickness of 14 μm, thaw-mounted onto conductive indium tin oxide (ITO) glass slides (Bruker Daltonics), and stored at -80°C. Sections were dried gently under a flow of nitrogen and desiccated at room temperature for 15 min, after which they were imaged optically using a photo scanner (Epson perfection V500). The samples were then coated with derivatization reagents, 2, 4-diphenylpyrylium tetrafluoroborate (DPP-TFB). Stock solution of DPP-TFB (8 mg in 1.2 ml MeOH) was prepared and diluted in 6 mL of 70% methanol containing 3.5 μL of trimethylamine. An automated pneumatic sprayer (TM-Sprayer, HTX Technologies, Carrboro, NC, United States) was used to spray DPP-TFB solution over the tissue sections. The nozzle temperature was set at 80°C and the reagent was sprayed for 30 passes over the tissue sections at a linear velocity of 110 cm/min with a flow rate of about 80 μL/min. Samples were then incubated for 15 min (dried by nitrogen flow every 5 min) in a chamber saturated with vapor from a 50% methanol solution. MALDI-MSI experiment was performed using a MALDI-TOF/TOF (Ultraflextreme, Bruker Daltonics, Bremen, Germany) mass spectrometer with a Smartbeam II 2 kHz laser in positive ion mode. The laser power was optimized at the start of each run and then held constant during the MALDI-MSI experiment.

### Data Analysis and Statistics

Autoradiograms from western blotting experiments were digitized using a Dia-Scanner (Epson Perfection 4870 PHOTO). Optical density values were measured using NIH Scion Image for Windows (alfa 4.0.3.2; © 2000–2001 Scion Corporation). Biochemical data were analyzed using one-way ANOVAs followed by Newman–Keuls *post hoc* test. Data from Amperometry were acquired and analyzed with the pClamp 9 or pClamp 10 software (Axon Instruments). Data are expressed as % of the baseline response measured for each slice during the 5–10 min preceding start of perfusion with T_1_AM. Statistical significance of the results was assessed by using Student’s *t*-test for paired observations (comparisons with baseline within single groups) or one-way ANOVA multiple comparison test followed by Newman–Keuls *post hoc* test since the samples from WT and KO mice were loaded in separated gels. The numbers of individual replicates are shown in the graphs, while *p*-values, degrees of freedom and *F* values are detailed in the results part.

## Results

### Dose Responses of Tyramine and T_1_AM on Phosphorylation of TH and GluA1 in Striatal Slices From TAAR1 Receptor WT and KO Mice

**FIGURE 1:**
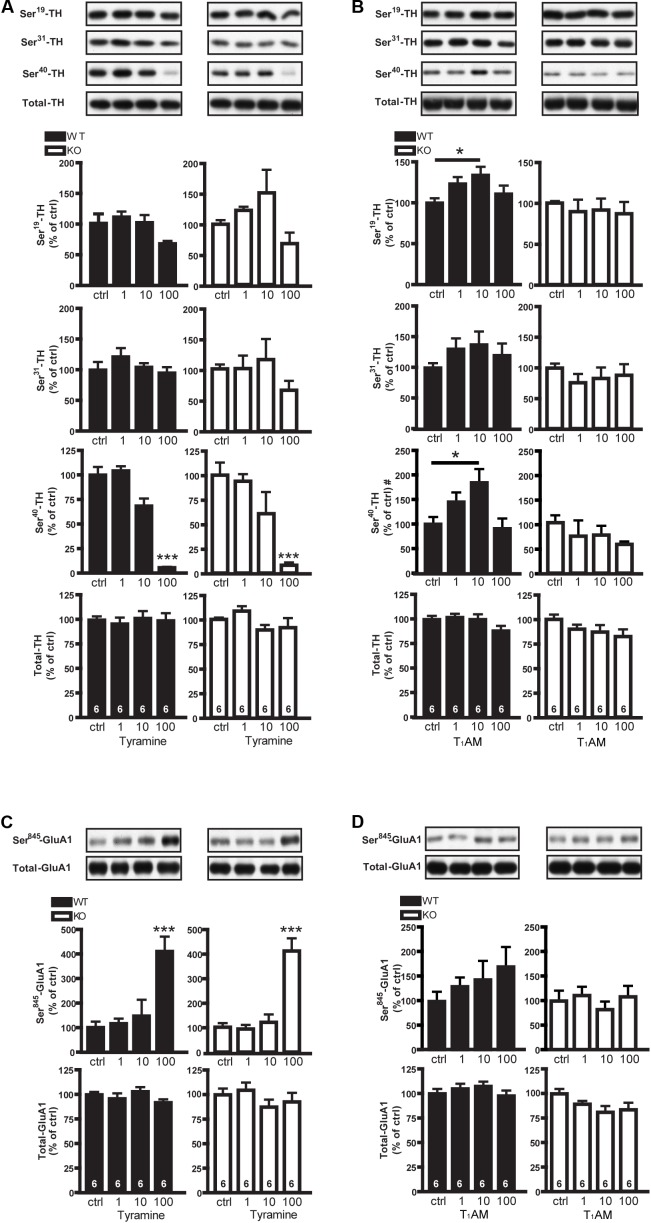
Effects of tyramine and T_1_AM on P-TH and P-GluA1 in striatal slices from WT and TAAR1 KO mice. Immunoblots against P-Ser^19^-TH, P-Ser^31^-TH, P-Ser^40^-TH, and total TH in control slices from TAAR1 WT and KO mice and in slices treated with tyramine (1, 10, 100 μM) **(A)** or T_1_AM (1, 10, 100 μM) **(B)**. Histograms show the quantifications of P-Ser^19^-TH, P-Ser^31^-TH, P-Ser^40^-TH, and total TH levels, respectively. Immunoblots against P-Ser^845^-GluA1 and total GluA1 in control slices from WT and TAAR1 KO mice and in slices treated with tyramine (1, 10, 100 μM) **(C)**, or with T_1_AM (1, 10, 100 μM) **(D)**. Histograms show the quantifications of P-Ser^845^-GluA1 and total GluA1 levels, respectively. Data were normalized to total protein levels. The images are parts of the same gels. ^∗^*p* < 0.05; ^∗∗∗^*p* < 0.001; one-way ANOVA followed by Newman–Keuls test for pairwise comparisons. The number of individual replicates is indicated within each column, ^#^denotes the number of individual replicates is 5 for each group of P-Ser^40^-TH.

We first studied the dose responses of the tyramine and T_1_AM on the phosphorylation of TH and GluA1 in striatal slices from TAAR1 receptor WT and KO mice. To study effects of compounds in the both genotypes, their individual baseline was set at 100%. One way ANOVA analysis showed that tyramine caused a significant change of P-Ser^40^-TH in both WT (*F*_[3,20]_: 60.28; *p* < 0.0001) and KO mice (*F*_[3,20]_: 9.928, *p* = 0.0003). *Post hoc* test showed that the lower (1 and 10 μM) concentrations of tyramine did not have any significant effect, whereas 100 μM tyramine significantly reduced phosphorylation of P-Ser^40^-TH in both groups of mice, suggesting an effect independent of TAAR1 (**Figure 1A**). Meanwhile, there were no effects at the same concentration of tyramine on P-Ser^19^-TH (WT: *F*_[3,20]_: 2.913; *p* = 0.06; KO: *F*_[3,20]_: 2.718; *p* = 0.07) and P-Ser^31^-TH (WT: *F*_[3,20]_: 0.9955; *p* = 0.42; KO: *F*_[3,20]_: 0.9157; *p* = 0.45) in neither WT nor KO mice (**Figure 1A**). One way ANOVA analysis in T_1_AM treated mice revealed that only in the WT but not in the KO mice, the drug affected significantly the levels of P-Ser^19^ (WT: *F*_[3,20]_: 3.641; *p* = 0.0004; KO: *F*_[3,20]_: 0.1953, *p* = 0.8983) and P-Ser^40^-TH (WT: *F*_[3,16]_: 4.393, *p* = 0.02; KO: *F*_[3,16]_: 0.823, *p* = 0.5001). *Post hoc* test showed that 10 μM T_1_AM enhanced P-Ser^19^ and P-Ser^40^-TH in the striatum of WT but not TAAR1 KO mice (**Figure 1B**). The effects of T_1_AM were biphasic with a further increase in drug concentrations resulting in less phosphorylation of TH. There was no effect of T_1_AM on P-Ser^31^-TH both in WT (*F*_[3,20]_: 0.7911, *p* = 0.5131) and KO mice (*F*_[3,20]_: 0.446, *p* = 0.7228) (**Figure 1B**). Tyramine altered significantly the phosphorylation of P-Ser^845^-GluA1 in WT (*F*_[3,20]_: 9.387; *p* = 0.0004) and KO mice (*F*_[3,20]_: 22.89; *p* < 0.0001) (**Figure 1C**). In contrast, T_1_AM had no effect on P-Ser^845^-GluA1 in striatal slices from either TAAR1 WT (*F*_[3,20]_: 0.8744; *p* = 0.4709) or KO mice (*F*_[3,20]_: 0.4367; *p* = 0.7295) (**Figure 1D**). These data suggest that tyramine and T_1_AM act differently on pre- and post-synaptic striatal targets.

**FIGURE 2:**
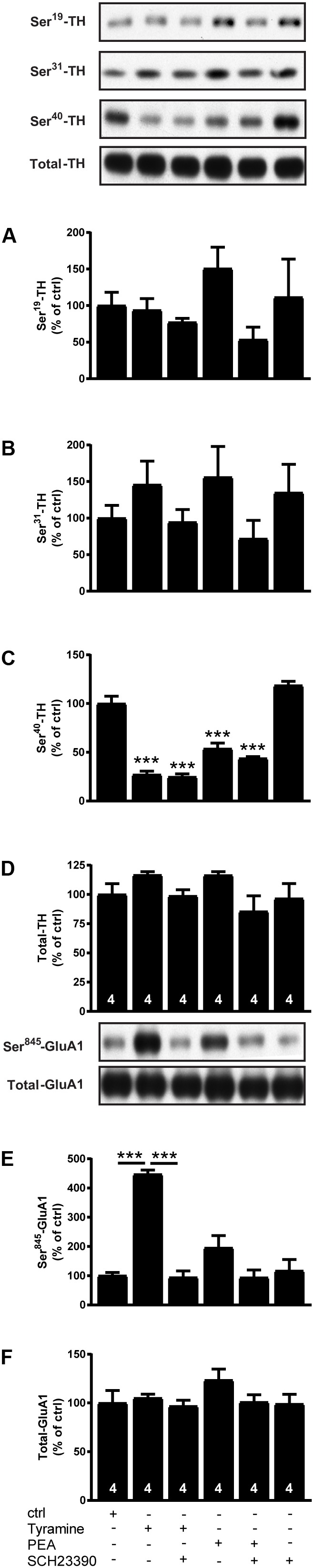
Effects of tyramine, β-PEA and SCH23390, alone or in combination, on P-TH and P-GluA1 in striatal slices from normal mice. Immunoblots against P-Ser^19^-TH, P-Ser^31^-TH, P-Ser^40^-TH, total TH, P-Ser^845^-GluA1, and total GluA1 in normal slices and in slices treated with tyramine (100 μM), β-PEA (100 μM) and SCH23390 (5 μM), alone or in combination. Histograms show the quantifications of P-Ser^19^-TH **(A)**, P-Ser^31^-TH **(B)**, P-Ser^40^-TH **(C)**, total TH **(D)**, P-Ser^845^-GluA1 **(E)**, and total GluA1 **(F)**, respectively. Data were normalized to total protein levels. ^∗∗∗^*p* < 0.001; one-way ANOVA followed by Newman–Keuls test for pairwise comparisons. The number of individual replicates is indicated within each column.

To further study the effect of a high dose of endogenous TAs, we incubated striatal slices from WT mice with the tyramine (100 μM), β-PEA (100 μM) alone or with the D_1_ receptor antagonist SCH23390 (5 μM). As shown in **Figure 2**, we found that β-PEA, like tyramine, decreased P-Ser^40^-TH (*F*_[5,18]_: 68.88; *p* < 0.0001) while not significantly affecting P-Ser^19^-TH (*F*_[5,18]_: 1.442; *p* = 0.2573) or P-Ser^31^-TH (*F*_[5,18]_: 1.18; *p* = 0.3574). The effects of tyramine and β-PEA on P-Ser^40^-TH were not affected by SCH23390. On the other hand, tyramine significantly enhanced P-Ser^845^-GluA1 (*F*_[5,18]_: 2.455; *p* < 0.0001), an effect that was reversed to baseline by D_1_ receptor blockade using SCH23390. Likewise, β-PEA tended to increase P-Ser^845^-GluA1, but this effect did not reach significance. The data of β-PEA was further to confirm that classical TAs and T_1_AM act differently.

### Effects of Tyramine and T_1_AM on TH Activity Measured by L-DOPA in Striatal Slices From TAAR1 WT and KO Mice

**FIGURE 3:**
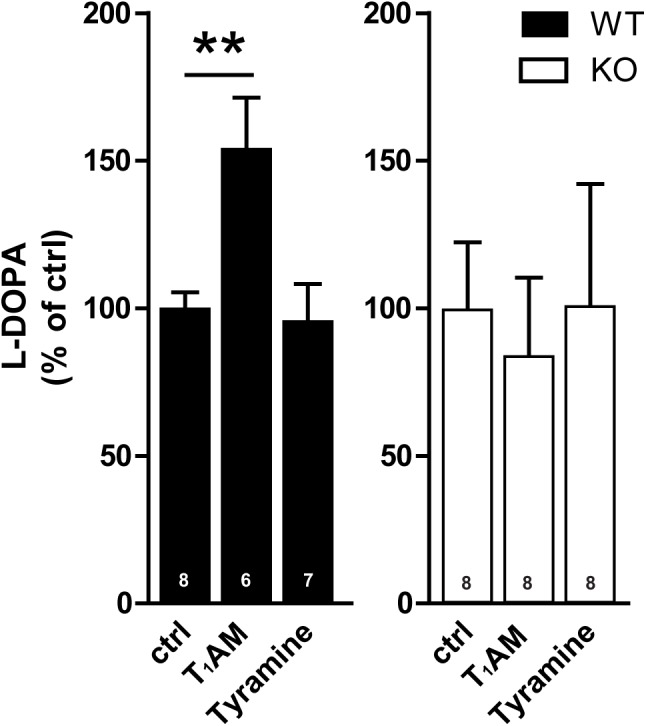
Effect of tyramine and T_1_AM on TH activity measured by L-DOPA in striatal slices from WT and TAAR1 KO mice. The activity of TH as measured by L-DOPA was enhanced by T_1_AM (10 μM) in WT mice whereas no change was detected in TAAR1 KO mice. Tyramine (100 μM) had no effect on L-DOPA. ^∗∗^*p* < 0.01; one-way ANOVA followed by Newman–Keuls test for pairwise comparisons. The number of individual replicates is indicated within each column.

There was also a baseline increase of TH activity in TAAR1 KO mice as compared to WT mice (Di Cara et al., 2011). To study effects of compounds in the both genotypes, their individual baseline was set at 100%. One way ANOVA revealed significant difference among the groups in WT mice (*F*_[2,18]_: 6.856; *p* = 0.0061) (**Figure 3**). *Post hoc* test showed that T_1_AM (10 μM), but not tyramine (100 μM), induced L-DOPA accumulation in the presence of a DOPA decarboxylase inhibitor in WT mice. No effects were detected in TAAR1 KO mice (*F*_[2,18]_: 0.08823; *p* = 0.9159), indicative of a TAAR1-mediated mechanism of action of T_1_AM.

### Effects of T_1_AM Alone or in Combination With H-89, 8-CPT-2Me-cAMP, and KN-92 on Phosphorylation of TH in Dorsal Striatal Slices From WT Mice

**FIGURE 4:**
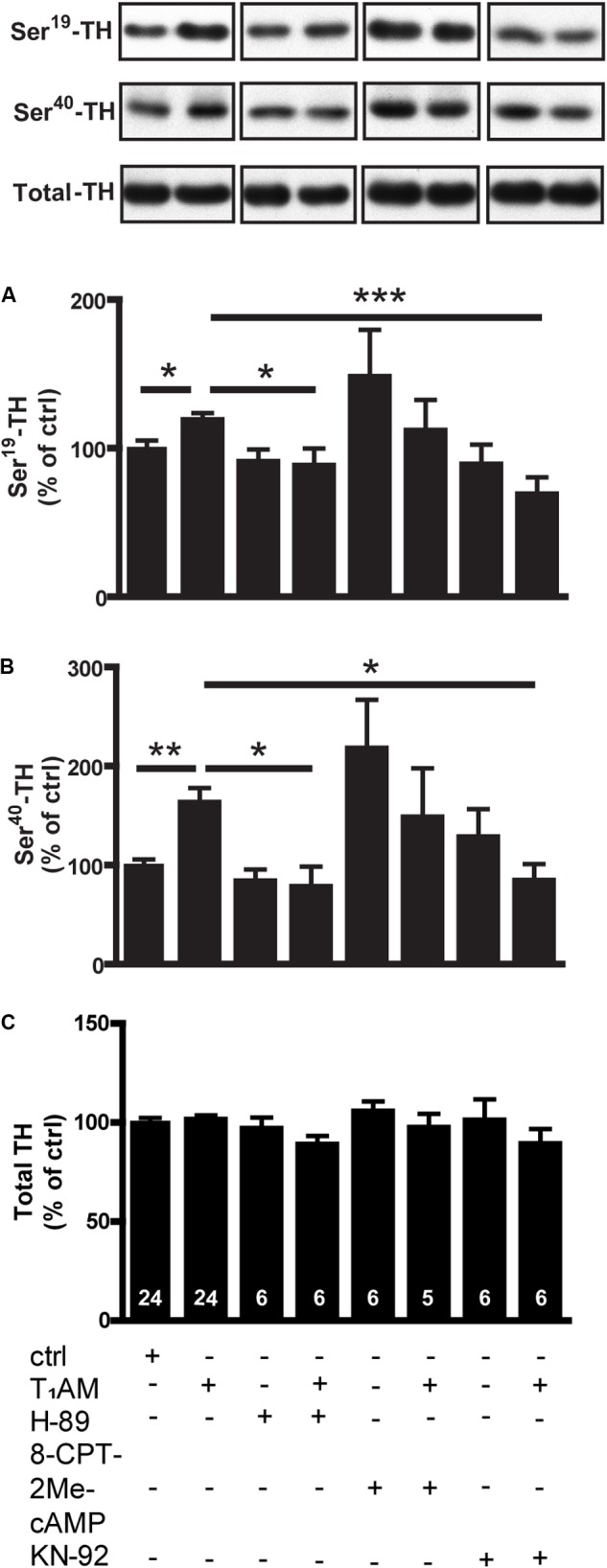
Effects of T_1_AM, H-89, 8-CPT-2Me-cAMP and KN-92, alone or in combination, on P-TH in striatal slices from normal mice. Immunoblots against P-Ser^19^-TH, P-Ser^40^-TH, and total TH in control slices and in slices treated with T_1_AM (10 μM), H-89 (10 μM), 8-CPT-2Me-cAMP (10 μM), and KN-92 (10 μM), alone or in combination. Histograms show the quantifications of P-Ser^19^-TH **(A)**, P-Ser^40^-TH **(B)**, and total TH **(C)** levels, respectively. Data were normalized to total level. The images are parts of the same gels. ^∗^*p* < 0.05, ^∗∗^*p* < 0.01, ^∗∗∗^*p* < 0.001; one-way ANOVA followed by Newman–Keuls test for pairwise comparisons. The number of individual replicates is indicated within each column.

To further study intracellular signaling cascades underlying the T_1_AM-induced phosphorylation of TH, we combined the T_1_AM with KN-92 or H-89, inhibitors of CamKII and protein kinase A (PKA), respectively. The effects of T_1_AM on both P-Ser^19^ (*F*_[7,75]_: 3.651; *p* = 0.0019) and P-Ser^40^-TH (*F*_[7,75]_: 3.871; *p* = 0.0012) could be significantly inhibited by either KN-92 or H-89 (**Figure 4**). Since TAAR1 is a Gs-coupled receptor and generates cAMP, we also examined the effects of 8-CPT-2Me-cAMP, an EPAC (Exchange Protein directly Activated by cAMP) activator, on TH phosphorylation. 8-CPT-2Me-cAMP alone tended to increase TH phosphorylation, but did not interact with T_1_AM (**Figure 4**).

### Effects of T_1_AM and EPPTB, Alone or in Combination, on Evoked Dopamine Release and Phosphorylation of TH in Dorsal Striatal Slices From WT and TAAR1 KO Mice

**FIGURE 5:**
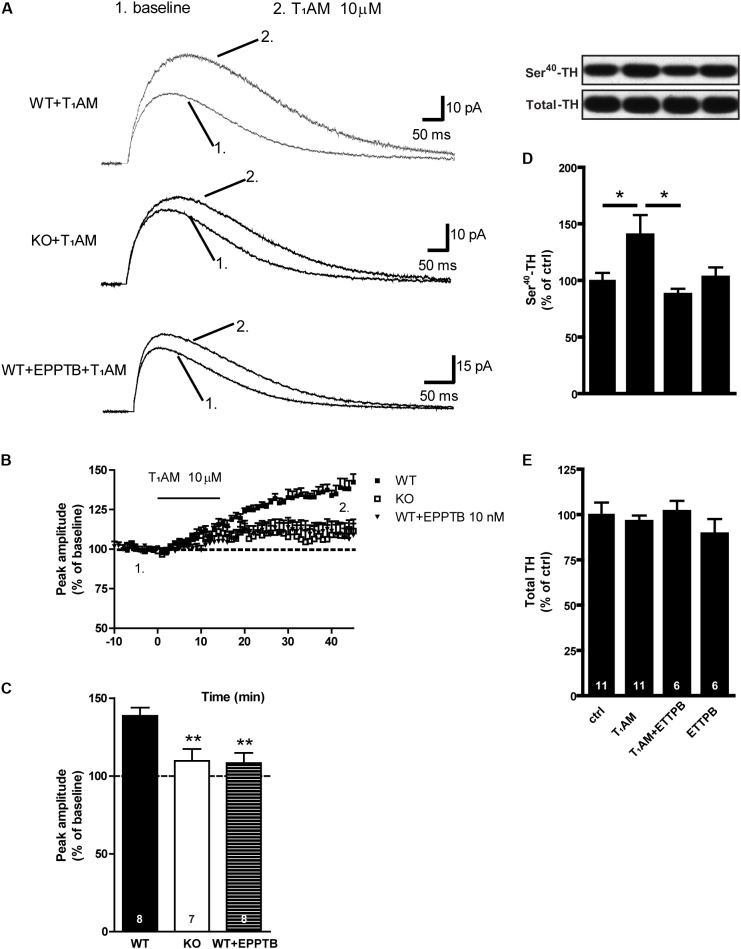
Effect of T_1_AM and EPPTB alone or in combination on evoked DA release and P-TH in in striatal slices from WT and TAAR1 KO mice. Representative traces from amperometric recordings in one slice before and after the application of T_1_AM (10 μM) in the perfusion solution from WT, TAAR1 KO, and EPPTB (10 nM) treated WT striatal slices, respectively **(A)**. Time course of the effect of T_1_AM on the normalized peak amplitude of evoked DA release measured with carbon fiber electrodes coupled to amperometry in striatal brain slices of WT, and TAAR1 KO mice, or with EPPTB **(B)**. Histograms show the quantifications of the last 5 min of recoding **(C)**. Immunoblots against P-Ser^40^-TH and total TH in control slices and in slices treated with T_1_AM (10 μM), EPPTB (10 nM). Histograms show the quantifications of P-Ser^40^-TH **(D)** and total TH **(E)** levels, respectively. Data were normalized to total level. ^∗^*p* < 0.05; ^∗∗^*p* < 0.01; one-way ANOVA followed by Newman–Keuls test for pairwise comparisons. The number of individual replicates is indicated within each column.

We evaluated the effect of T_1_AM on stimulation-evoked release of DA from DA-containing fibers present in sagittal striatal slices, as shown in **Figure 5**. We found that bath application of T_1_AM (10 μM) significantly increased the amplitude of evoked DA release measured with carbon fiber electrodes coupled to amperometry in dorsal striatal brain slices from WT mice, and that this effect was significantly reduced in TAAR1 KO mice. In presence of the TAAR1 antagonist EPPTB (10 nM), the effect of T_1_AM on evoked DA release in WT mice was significantly reduced, but not completely blocked (*F*_[2,20]_: 7,252; *p* = 0.0043). EPPTB had no effect on evoked DA release by itself (data not shown). Similar to the results as above, T_1_AM significantly increased P-Ser^40^-TH (*F*_[3,30]_: 3.384; *p* = 0.0309). This effect was blocked when T_1_AM was combined with EPPTB, suggesting a mechanism of action mediated via TAAR1.

### The Relative Distribution and Abundance of T_1_AM in Sagittal Brain Sections From WT and TAAR1 KO Mice

**FIGURE 6:**
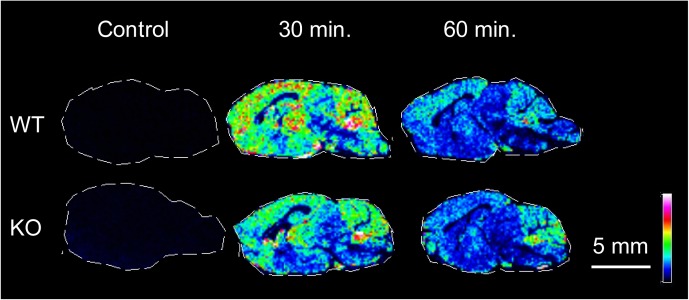
The relative distribution and abundance of T_1_AM, derivatized by DPP-TFB are acquired on sagittal tissue sections from TAAR1 WT and KO mice. No significant signal was detected on controls while signals correspond to derivatized T_1_AM (m/z 623.1) was detected in administered animals at 30 and 60 min post-dose (20 mg/kg). The concentration of the drug appeared higher in both genotypes after 30 min compared to 60 min post-dose. MS images were acquired using a MALDI-TOF/TOF mass spectrometer. Data are shown using a rainbow scale, normalized against the total ion count. Scale bar, 5 mm; spatial resolution = 150 μm.

T_1_AM was derivatized by 2, 4 diphenyl pyranylium and detected by MALDI-MSI. No clear endogenous signal of T_1_AM was found in uninjected sections neither from WT nor TAAR1 KO mice. However, widespread signals corresponding to derivatized T_1_AM was detected in mice intraperitoneally administered with T_1_AM (20 mg/kg). The concentration of the drug appeared higher after 30 min compared to 60 min post-dose (**Figure 6**).

## Discussion

Our experiments demonstrate that the thyronamine T_1_AM enhance phosphorylation and activity of TH along with evoked DA release in dorsal striatum, while not significantly affecting the phosphorylation of post-synaptic AMPA receptor GluA1 subunits. The effects on TH phosphorylation observed following T_1_AM administration were abolished in TAAR1 KO mice, while the effects on evoked DA release were attenuated in TAAR1 KO mice and following TAAR1 blockade, supporting a role of TAAR1 as a partial mediator of these effects. We can conclude that T_1_AM acts through TAAR1 to enhance the production of dopamine. This *de novo* dopamine creation heightens the synaptic dopamine content and raises extracellular dopamine. In contrast, tyramine and β-PEA reduced TH phosphorylation via a mechanism independent of TAAR1.

### The Differential Effects of the TAs Tyramine, β-PEA and Thyronamine, T_1_AM, on Phosphorylation and Activity of TH and on GluA1 Phosphorylation

**FIGURE 7:**
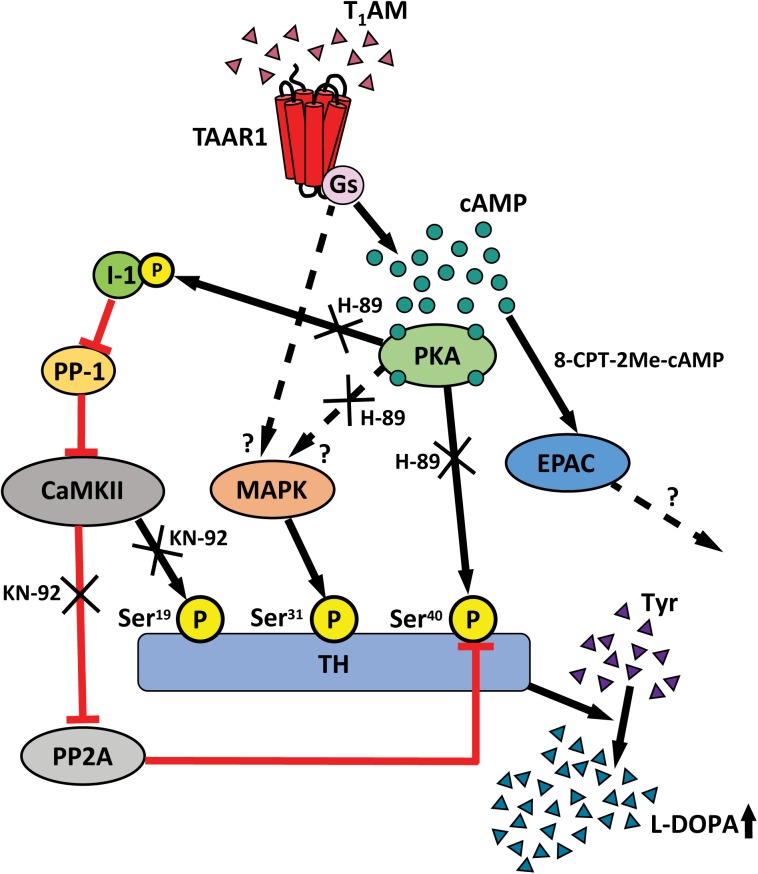
Schematic graph show the cellular signaling pathway for TAAR1 acting on P-TH. TAAR1 is Gs-coupled receptor and activation of TAAR1 results in stimulating cAMP, PKA that can directly activate the phosphorylation of Ser^40^-TH. Another pathway involves PKA that activate P-I-1, which in turn inhibits protein phosphatase 1 (PP-1). PP-1 inhibits the phosphorylation of CamKII which active phosphorylation of Ser^19^-TH. CamKII can also inhibit protein phosphatase 2A (PP2A) which activates phosphorylation of Ser^40^-TH. This higher phosphorylation rate leads to the accumulation of DOPA.

Alterations of DA synthesis are regulated via phosphorylation of TH, the rate-limiting enzyme in the synthesis of catecholamines (Daubner et al., 2011), and Ser^19^, Ser^31^, and Ser^40^ have been identified as the functionally most important sites of TH phosphorylation (Haycock and Haycock, 1991). Phosphorylation of Ser^19^ is induced by enhanced intracellular Ca^2+^ concentrations and activation of CaM kinase II, whereas phosphorylation at Ser^31^ is induced by extracellular signal-regulated protein kinases, and phosphorylation of Ser^40^ is catalyzed by PKA (Haycock, 1993). TH phosphorylation at Ser^40^ and Ser^31^ leads to increased TH activity, whereas phosphorylation at Ser^19^ exerts a positive modulatory influence on Ser^40^ phosphorylation (Bobrovskaya et al., 2004; Dunkley et al., 2004). Notably, TAAR1 KO animals exhibit a basal increase in TH activity and increased basal phosphorylation at Ser^19^, Ser^31^, and Ser^40^ in striatal slices compared to WT animals, perhaps due to developmental compensations (Di Cara et al., 2011). An increased TH activity at Ser^19^, Ser^31^, and Ser^40^ was observed in WT mice following administration of 1 to 10 μM T_1_AM, but this effect was attenuated upon increasing or lowering the concentration, indicating a bell-shaped dose–response. The effects of T_1_AM on TH activity were abolished in TAAR1 KO mice, identifying TAAR1 as a mediator of these actions. TAAR1 is coupled with stimulatory G_s_ proteins as well as G protein independent pathways and, upon activation, TAAR1 signals through the cAMP/PKA/CREB, β-arrestin2/Akt/GSK-3β and the protein kinase C (PKC)/Ca++/NFAT pathways (Borowsky et al., 2001; Bunzow et al., 2001; Panas et al., 2012; Harmeier et al., 2015). Here we found that the T_1_AM-mediated increases on Ser^19^ and Ser^40^ TH were inhibited by blockade of CamKII and PKA by either KN-92 or H-89, respectively. The protein responsible for the phosphorylation of TH at the site Ser^31^ is MAPK (Haycock et al., 1992). As a consequence we suppose that the TAAR1’s activation by T_1_AM stimulates the activity of MAPK, either through PKA or through an alternative direct pathway like β-arrestin2 (Pierce and Lefkowitz, 2001). In order to confirm that the observed phosphorylation of TH leads to increased enzymatic activity, we made measurements of DOPA with HPLC in striatal slices. T_1_AM induced a higher level of DOPA accumulation in the presence of a DOPA decarboxylase inhibitor in WT mice. This effect of T_1_AM was abolished in KO counterparts. **Figure 7** shows a schematic, and somewhat speculative, drawing of the proposed signaling pathway induced by T_1_AM/TAAR1/PKA activation. TAAR1 is a G_s_ protein-coupled receptor and activation results in increased cAMP via activation of adenylyl cyclase and PKA signaling, which directly phosphorylates TH at Ser^40^. Another pathway could involve activation of inhibitor 1 by PKA, which inhibits protein phosphatase 1 (PP-1). PP-1 inhibits the phosphorylation of CamKII, which activates phosphorylation of Ser^19^-TH. CamKII can also inhibit protein phosphatase 2A which activates phosphorylation of Ser^40^-TH. The regulation of the suggested signal transduction pathways could explain how KN-92 and H-89, inhibitors of CamKII and PKA respectively, both can block the effect of T_1_AM. 8-CPT-2Me-cAMP, the EPAC analog, had no influence on TH phosphorylation. In summary, this higher phosphorylation rate leads to the accumulation of DOPA.

In contrast to T_1_AM, under the present conditions, the endogenous TAs tyramine and β-PEA appeared to elicit mainly non-TAAR1 dependent effects. Tyramine, at a high dose, reduced phosphorylation of Ser^40^-TH, but increased phosphorylation of the post-synaptic AMPA receptor GluA1 subunit in striatal slices from both WT and TAAR1 KO mice. Similarly, another endogenous TA, β-PEA also reduced phosphorylation of Ser^40^-TH and tended to increase phosphorylation of Ser^845^-GluA1 in slices from WT mice. The quantification of GluA1 phosphorylation on Ser^845^, help us to evaluate the status of GluA1 in the post-synaptic membrane of corticostriatal and thalamostriatal synapses, which are the main classes of glutamatergic synapse in the striatum (Smith et al., 2004). The corticostriatal and thalamocortical projection neurons innervate the principal population of MSNs, but also various subtypes of interneurons (Smith et al., 2004). By measuring the phosphorylation of GluA1 we cannot rule out which types of interneurons and MSNs (D_1_ positive or D_2_ positive) are activated. Nevertheless, it is shown that D_1_ agonists and D_2_ antagonists induce robust increases in GluA1 phosphorylation while D_2_ agonists and D_1_ antagonists have no effect (Xue et al., 2017). Considering this fact, we deduce that the effect of tyramine on GluA1 trafficking could be explained by either the post-synaptic regulation of D_1_ or D_2_ receptors. D_1_ receptor blockade by SCH23390 blocked the tyramine-induced phosphorylation of GluA1 subunits, but had no effect on tyramine- or β-PEA-reduced Ser^40^-TH phosphorylation, suggesting that the post-synaptic effects of tyramine are dependent on D_1_-receptor activation. According, GluA1 upregulation may be a consequence of the dopamine’s net effect upon D_1_ and D_2_ receptors (Xue et al., 2017). In our study, we observed that tyramine attenuated the TH phosphorylation at Ser^40^. However, diminished enzymatic activity of TH does not mean a reduction in dopamine release, whilst a negative feedback mechanism for dopamine control of TH activity has been documented (Lindgren et al., 2001; Daubner et al., 2011). It is conceivable that tyramine has amphetamine-like effects on the excitability of the post-synaptic membrane and possibly leads to the vesicular leak of dopamine by its interaction with VMAT2 (Zhu et al., 2007). Moreover, it has been suggested that TAs act like amphetamines and could increase extracellular DA levels by promoting DA release via inducing reversal of the dopamine transporter (DAT) and by displacing DA from vesicular stores (Sulzer et al., 1995; Jones et al., 1998; Mundorf et al., 1999). β-PEA, which is structurally related to amphetamine, has been proposed to act as an endogenous amphetamine (Janssen et al., 1999), and has previously been shown to increase extracellular levels of DA in striatum and NAc via a DAT-dependent mechanism (Sotnikova et al., 2004; Murata et al., 2009). Notably, Xie and Miller found that TAs, including tyramine and β-PEA, do not directly activate monoamine autoreceptors (Xie and Miller, 2008). However, they have been proposed to indirectly activate dopamine autoreceptors by enhancing the efflux of dopamine (Geracitano et al., 2004). One possible explanation for tyramine’s TAAR1 independent effect on GluA1 phosphorylation, may be that this TA acts through MSN-localized TAAR1 to affect the availability of GluN1 and through VMAT2 to alter the surface density of GluA1. Indeed, several studies have supposed that tyramine can affect glutamate receptor membrane availability through MSN-localized TAAR1 (Alvarsson et al., 2015; Espinoza et al., 2015a; Sukhanov et al., 2016). To conclude, our data support the notion that T_1_AM can modulate DA synthesis via a mechanism of action that involves presynaptic TAAR1. Moreover, we suppose a direct effect of tyramine on dopamine release that could lead to the observed decline in TH phosphorylation due to secondary activation of indirect D_2_ autoreceptors (Lindgren et al., 2001).

### The Effect of T_1_AM on Evoked DA Release in the Dorsal Striatum Using Amperometry

Our experiments suggest that striatal dopamine release can be enhanced by T_1_AM-mediated TAAR1 activation. However, most previous slice experiments addressing the modulatory influence of TAAR1 on the dopaminergic system have been performed in the VTA (Lindemann et al., 2008; Revel et al., 2012), which may be a source of discrepancy between our study and previous findings. Previous slice experiments in the VTA of TAAR1 KO mice revealed enhanced spontaneous firing rates of dopaminergic neurons in TAAR1 KO mice compared to WT mice, suggesting that TAAR1 exerts an attenuating effect on dopaminergic neuron activity (Lindemann et al., 2008). This has been supported by slice experiments in mouse VTA using the specific ligands RO5166017 and EPPTB to stimulate and block TAAR1, respectively (Bradaia et al., 2009; Revel et al., 2011). However, the increased DA neuron firing rate observed in TAAR1 KO mice did not lead to enhanced basal levels of extracellular striatal DA compared to WT mice as detected by microdialysis (Lindemann et al., 2008). Indeed, mice overexpressing TAAR1, like TAAR1 KO mice, also exhibit an enhanced spontaneous firing activity of monoaminergic neurons of the VTA, DRN, and locus coeruleus (Revel et al., 2012). Moreover, it is likely that the functional outcome of TAAR1 activation differs between specific classes of ligand and distinct brain regions depending on the characteristics of the dopaminergic innervation and basal tone. Although midbrain DA neurons are considered to be relatively homogenous, emerging data support a high level of diversity among VTA and SNc neurons as regards electrophysiological properties, synaptic connectivity, protein expression profiles, and behavioral functions (Roeper, 2013). The expression levels of two TAAR1 related proteins, D_2_ receptor and GIRK2, are implicated in the differences between the two subpopulations (Bradaia et al., 2009; Reyes et al., 2012; Bifsha et al., 2014; Brichta and Greengard, 2014). In agreement with this, it is reported that TAAR1 has a differential role in dopamine release between VTA and SNc projection sites in striatum (Leo et al., 2014). In contrast, Di Cara et al. (2011) showed that TAAR1 decreases the amplitude of Methylenedioxymethamphetamine (MDMA) induced dopamine release both in ventral and dorsal striatum. In the same study it was observed that the TAAR1 agonist, *o*-phenyl-3-iodotyramine (*o*-PIT) blunted the para-chloroamphetamine (PCA) induced dopamine release in both structures (Di Cara et al., 2011). Accordingly, TAAR1 may exert a complex pattern of effects on dopaminergic terminals in ventral as compared to dorsal compartments of the striatum. Furthermore, both the VTA and SNc can be further distinguished regarding the expression of calbindin D28k (CB), with the highest density of CB positive neurons located in VTA (Surmeier et al., 2017). CB positive neurons have the tendency to send projections in CB poor islands in striatum (striosomes), while CB negative cells mainly innervate CB rich regions of the striatum (striatal matrix) (Brimblecombe and Cragg, 2017; Sgobio et al., 2017). It has been reported that the evoked striatal DA release differs between these two compartments but also that the dopamine release ratio of striosome over matrix is higher in the ventral than dorsal striatum (Salinas et al., 2016). Consequently, TAAR1 could have diverse effects not only among VTA and SNc neurons but also between CB positive and negative subgroups.

Our findings with T_1_AM, may also be explained by differences in the methodologies and protocols employed to evoke and measure dopamine release. For example, the use of strong stimulation intensities might evoke maximal release. Conversely, local, low intensity stimulation, as used in the present study, allows for observation of both inhibition and potentiation of dopamine release. In addition, recent studies have demonstrated that dopamine release in brain slices can be evoked by direct stimulation of dopaminergic axons and indirectly by stimulation of cholinergic interneurons in the striatum (Threlfell et al., 2012; Zhang et al., 2014b). It has not yet been established whether cholinergic neurons express TAAR1 but the contrasting effects of RO5166017 and T_1_AM might result from differences in the involvement of cholinergic control of dopamine release between different experimental paradigms. Taken together, these studies raise the question of a possible differential control of dopamine release by TAAR1 receptors in cholinergic interneurons and in dopamine axon terminals.

In this study, we investigated the action of T_1_AM at TAAR1 on dopaminergic terminals as compared to those of TAs. However, T_1_AM is also known to be an agonist of TAAR5 (Dinter et al., 2015c). Moreover, the β-phenylethylamine-like structure affords T_1_AM the ability to bind with various members of GPCR superfamily and ion channels (Chiellini et al., 2017; Khajavi et al., 2017). It is indeed claimed that T_1_AM interacts with α2a adrenergic receptors, β2-adrenergic receptors and muscarinic receptors (Kleinau et al., 2011; Dinter et al., 2015a,b; Laurino et al., 2016, 2017). Notably, outside the CNS, T_1_AM has been found to differentially regulate insulin secretion through actions at TAAR1 and α2a adrenergic receptor (Chiellini et al., 2017; Lehmphul et al., 2017). Hence, despite blockade of the actions of T_1_AM in KO mice and by pharmacological antagonist, the possibility that it exerts actions via other mechanisms should not be excluded.

In this study we incubated the slices in a T_1_AM containing buffer. It is important to access the roles of T_1_AM in the intact brain. We show here that T_1_AM can be detected by MALDI-MSI in mouse brain slices 30 and 60 min after systemic administration. Since T_1_AM was detected in many brain areas, we can conclude that T_1_AM can penetrate the blood brain barrier. This finding is in accordance with previous studies showing effects on glucose metabolism by intraperitoneally administered T_1_AM (Klieverik et al., 2009). Using MALDI-MSI, no clear endogenous levels of T_1_AM could be detected. However, it will be interesting to study T_1_AM levels in pathological states, particularly in hyperthyroid conditions. In addition to a circulating source, direct enzymatic transformation of T_4_ to T_1_AM may occur in neurons. The responsible enzyme for this reaction is ornithine decarboxylase (Hoefig et al., 2016), which is expressed by neuronal and astroglial cell types of the CNS (Bernstein and Müller, 1999). Apart from T_1_AM itself, its metabolite 3-iodothyroacetic acid (TA1) is implicated in the modulation of histaminergic neurotransmission and might likewise interact with dopaminergic pathways: this remains to be clarified (Laurino et al., 2015).

## Conclusion

This study demonstrates that TAAR1 mediates the effects of T_1_AM on dorsal striatal TH phosphorylation, activity and evoked dopamine release. No comparable alterations were found after application of tyramine and β-PEA. This simultaneous augmentation in TH phosphorylation and striatal dopamine release after the administration of T_1_AM indicates that this thyronamine favors dopamine synthesis and subsequent secretion through TAAR1. Conversely, TAs act in a TAAR1 independent manner to influence dopamine secretion resulting in feedback inhibition of TH. This study further indicates that the modulatory properties of TAAR1 may differ depending on the identity of the ligand in question, the extracellular milieu, basal levels of monoamines, neuronal circuitry, and the cellular localization of TAAR1, which are mutually regulated by interactions with D_2_ receptors and DAT, and by the available signaling transduction systems. Further elucidation of the complex pattern of influence of TAAR1 upon monoaminergic and other pathways controlling mood, motor function and cognition may lead to the elaboration of urgently-need, novel strategies for improving the treatment of depression, schizophrenia, Parkinson’s disease, and other neuropsychiatric disorders (Millan et al., 2015; Khan and Nawaz, 2016; Berry et al., 2017; Cichero and Tonelli, 2017).

## Author Contributions

Participated in research design: XZ, KC, and PS. Collected the samples and conducted the experiments: XZ, MS, MP, AN, and TY. Performed the data analysis and discussed the data: XZ, IM, AA, TY, JK, PEA, MJM, KC, and PS. Contributed to the writing of the manuscript and to revising it critically for scientific discussions: XZ, IM, AA, MJM, KC, and PS. All authors approved the final version to be published.

## Conflict of Interest Statement

The authors declare that the research was conducted in the absence of any commercial or financial relationships that could be construed as a potential conflict of interest.
